# MEKK4-mediated Phosphorylation of HOXA10 at Threonine 362 facilitates embryo adhesion to the endometrial epithelium

**DOI:** 10.1038/s41420-022-01203-1

**Published:** 2022-10-10

**Authors:** Mei Zhang, Qun Zhang, Zhiwen Cao, Xinyu Cai, Jingyu Liu, Yue Jiang, Yingchun Zhu, Jidong Zhou, Lina Yu, Xin Zhen, Yali Hu, Guijun Yan, Haixiang Sun

**Affiliations:** 1 Center for Reproductive Medicine and Obstetrics and Gynecology, Nanjing Drum Tower Hospital, Nanjing University Medical School, Nanjing, 210008 China; 2 Center for Molecular Reproductive Medicine, Nanjing University, Nanjing, 210008 China; 3 State Key Laboratory of Pharmaceutical Biotechnology, Nanjing University, Nanjing, 210008 China; 4 State Key Laboratory of Reproductive Medicine, Nanjing Medical University, Nanjing, 211116 China

**Keywords:** Phosphorylation, Infertility

## Abstract

Embryo adhesion is a very important step in the embryo implantation process. Homeobox A10 (HOXA10), a key transcriptional factor of endometrial receptivity, is indispensable for embryo adhesion. However, how to control the activation status of HOXA10 remains elusive. Here, we found that Mitogen-activated protein kinase kinase kinase 4 (MEKK4) was associated with HOXA10 and directly phosphorylated HOXA10 at threonine 362. This MEKK4-mediated phosphorylation enhanced HOXA10-mediated transcriptional responses and adhesion between the embryo and endometrial epithelium. Specific deletion or kinase inactivation of MEKK4 in endometrial epithelial cells attenuates adhesion between embryo and epithelium. Therefore, the identification of MEKK4 as a novel physiological positive regulator of HOXA10 activation provides mechanistic insights to improve embryo implantation success. Moreover, when Thr362 was mutated to alanine (T362A) to mimic its dephosphorylation, the protein stability and transcriptional regulation of HOXA10 were decreased. In addition, HOXA10 -promoted embryo adhesion was weakened after the mutation of Thr362, suggesting that the phosphorylation of HOXA10 at this site may be a new indicator for evaluating endometrial receptivity and judging the ‘implantation window’.

## Introduction

Seventy-five percent of conception failures are due to embryo implantation failure [1].﻿ Successful embryo implantation requires a receptive endometrium, a viable embryo, and a synchronous dialog between the mother and the embryo. Developments in embryology and related culture techniques over the past 40 years have significantly improved embryo viability and the overall success rates of in vitro fertilization (IVF) [2]. Two-thirds of the reasons for embryo implantation failure are due to endometrial factors [1]. Recurrent implantation failure (RIF) is a typical example of a situation in which where endometrial factors may be the main cause of implantation failure following high-quality embryo transfer [3–5].

Multiple approaches are emerging to find reliable biomarkers to predict endometrial status to improve pregnancy outcomes [6]. Studies have shown that abnormal expression of several molecules, such as cytokines [7], homeobox genes [8], and cell adhesion molecules [9], are associated with infertility and miscarriage. The endometrial epithelium is the first part of the embryo to come into contact with the mother at implantation, and its structural and functional alterations are crucial for establishing endometrial receptivity and successful embryo implantation [10]. The transcription factor HOXA10 is known to play a vital role in regulating changes in epithelial cell function during the establishment of endometrial receptivity [11]. HOXA10 transcriptionally activates or represses the expression of multiple receptivity-related genes [12], such as ITGB3, which acts as an adhesion medium between the embryo and epithelial cells [13]. Clinical studies have indicated that reduced HOXA10 expression in uterine tissues is closely associated with embryo implantation failure in patients with hydrosalpinx [14], adenomyosis [15], endometriosis [16], and unexplained infertility [17]. Both posttranscriptional modifications (m6A methylation [18]) and posttranslational modifications (acetylation [19], sumoylation [20], and degradation [12]) of HOXA10 lead to impaired embryo adhesion. Some studies report that HOXA10 protein expression is reduced in the endometrium of RIF patients [8, 21], but our team found that the HOXA10 protein expression was not distinctly reduced in the endometrium of some RIF patients relative to normal controls [20]. Therefore, we remain focused on the novel regulatory mechanisms of HOXA10 to seek to provide mechanism-based insights into gene expression regulation in the process of embryo adhesion.

Phosphorylation modifications are the most prevalent posttranslational modifications in eukaryotic cells and play important functions in the regulation of protein self-stability, activity, and subcellular localization [22]. We identified that Mitogen-activated protein kinase kinase kinase 4 (MEKK4) exhibited a high probability of interacting with HOXA10 by using the yeast two-hybrid technique. MEKK4 is a serine/threonine kinase of the mitogen-activated protein kinase family, which regulates cell proliferation, apoptosis, and differentiation through activating the p38 and JNK signaling pathways [23], and these pathways, in turn, regulate neural tube development, bone formation [24], sex differentiation [25], and placenta formation [26].﻿

In the present study, we demonstrated the functional significance of MEKK4-mediated HOXA10 phosphorylation in upregulating the expression of ITGB3 protein, a downstream target of HOXA10. HOXA10 undergoes phosphorylation modification after estradiol and progesterone stimulation. Our study reveals that the aberrant expression of MEKK4 and phosphorylation of HOXA10 might have an influence on embryo adhesion and might contribute to the development of RIF.

## Results

### Estrogen and progesterone promote HOXA10 protein phosphorylation expression in endometrial epithelial cells

**Fig. 1:**
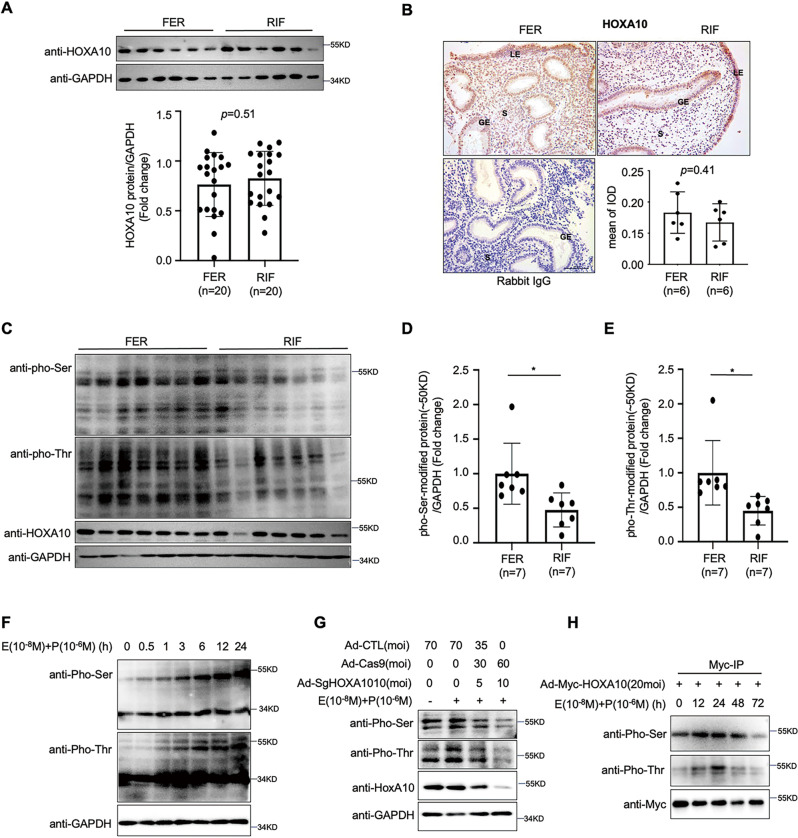
Estrogen and progesterone promote HOXA10 protein phosphorylation in endometrial epithelial cells. **A** Western blotting analysis of HOXA10 expression in the endometria of normal control women (*n* = 20) and infertile women with RIF (*n* = 20). HOXA10 protein levels were normalized to GAPDH protein expression levels to quantify the densitometric analysis results. Data are represented as mean ± SEM, using *t*-test. **B** Immunohistochemical analysis of the HOXA10 expression in endometria from fertile women (*n* = 6) and RIF patients (*n* = 6). Rabbit IgG was used as a negative control. The IOD of HOXA10 expression was calculated with Image-Pro Plus (200× magnification; scale bar = 100 µm). **C** Western blotting analysis of HOXA10, pho-Ser, and pho-Thr expression in endometria from normal control women (*n* = 7) and infertile women with RIF (*n* = 7). **D**, **E** pho-Ser and pho-Thr protein (~50 KD) levels were normalized to the GAPDH protein expression levels. Data are represented as mean ± SEM, *P < 0.05, using *t*-test. **F** Western blotting analysis of pho-Ser, and pho-Thr expression in Ishikawa cells treated with E_2_ and MPA for various times as indicated. **G** Western blotting analysis of HOXA10, pho-Ser, and pho-Thr expression in Ishikawa cells infected with the adenoviruses Ad-cas9 and Ad-SgHOXA10 were treated with E_2_ and MPA for 24 h. **H** Combination of Myc-IP with Western blotting analysis of pho-Ser, and pho-Thr expression in Ishikawa cells stimulated with the adenovirus Ad-Myc-HOXA10 and then treated with E_2_ and MPA for 24 h.

First, we investigated the endometrial expression of the HOXA10 protein, which was shown to be expressed at similar levels in the control fertile women and RIF patients (Fig. 1A–C). The serine/threonine phosphorylation level of the total endometrial proteins of RIF patients showed a downward trend, and the band near the position of the HOXA10 protein (according to its molecular weight of ~50 KD) was significantly reduced (Fig. 1C–E). Then, we found that serine/threonine phosphorylation levels of total protein increased at 24 h after the estrogen and progesterone stimulation of endometrial epithelial Ishikawa cells, especially at the ~50 KD position (Fig. 1F). After silencing endogenous HOXA10 expression in Ishikawa cells, the serine/threonine phosphorylation band located around the molecular weight of the HOXA10 protein gradually diminished with the decrease in HOXA10 protein expression after 24 h of estrogen and progestin stimulation (Fig. 1G). In addition, high HOXA10 expression was achieved in Ishikawa cells, mediated by the adenovirus Ad-myc-HOXA10, and immunoprecipitation results showed that the serine phosphorylation level of the HOXA10 protein increased from 12 h of estrogen and progesterone stimulation, and while the highest level of threonine phosphorylation was observed at 24 h (Fig. 1H). Therefore, the HOXA10 protein in endometrial epithelial cells is modified by phosphorylation under estrogen and progesterone stimulation.

### MEKK4 is a novel interactor of HOXA10

**Fig. 2:**
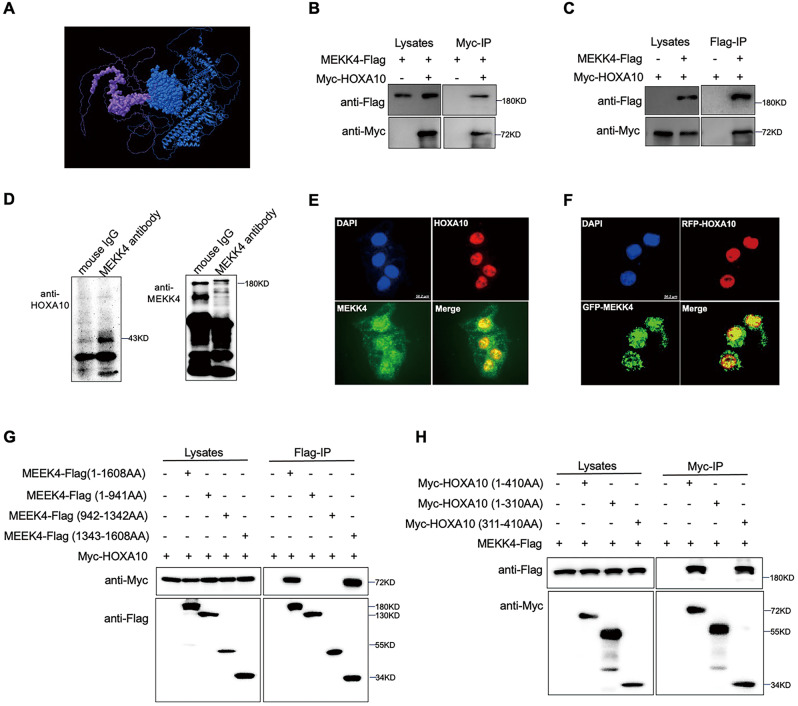
Interaction between HOXA10 and MEKK4. **A** The MEKK4-interacting protein HOXA10 was screened with Python software based on data modeling and protein structure prediction. (Blue, MEKK4; bold blue, MEKK4 kinase domain; Yellow, HOXA10; bold yellow, HOXA10 homeobox). **B**, **C** Combination of exogenous Flag-IP or Myc-IP with Western blotting analysis of the interaction between HOXA10 and MEKK4 in 293 T cells co-transfected with Myc-HOXA10 and MEKK4-Flag for 48 h. **D** Combination of exogenous anti-MEKK4 endogenous immunoprecipitation with Western blotting analysis of the interaction between HOXA10 and MEKK4 in Ishikawa cell lysates. Mouse IgG was used as a negative control. **E** Cellular localization of endogenous MEKK4 and HOXA10 in Ishikawa cells. (Scale bar = 56 µm) **F** Cellular localization of GFP-MEKK4 and RFP-HOXA10 in Ishikawa cells. (Scale bar = 56 µm) **G** Combination of exogenous Flag-IP with Western blotting analysis of the interaction between HOXA10 and MEKK4 mutant constructs in 293 T cells. **H** Combination of exogenous Myc-IP with Western blotting analysis of the interaction between MEKK4 and HOXA10 mutant constructs in 293 T cells.

The potential MEKK4-interacting protein HOXA10 was screened with Python software based on data modeling and protein structure prediction (Fig. 2A). We next examined whether MEKK4 interacted with HOXA10. In 293 T cells, MEKK4-Flag specifically interacted with Myc-HOXA10 (Fig. 2B, C). In Ishikawa cells, endogenous MEKK4 could also interact with HOXA10 proteins (Fig. 2D). In addition, nuclear colocalization of two proteins, endogenous and exogenous, was presented in Ishikawa cells (Fig. 2E, F). Furthermore, full-length HOXA10 bound the C-terminus of MEKK4(Fig. 2G), which contains the kinase domain, and full-length MEKK4 coprecipitated with the C-terminus of HOXA10 (Fig. 2H), which includes the homeobox, a highly conserved helix-loop-helix (HTH) domain that recognizes TTAT or TAAT sequences in the promoter region of downstream target genes and regulates their transcriptional activation or inhibition [27]. This confirmed the software prediction in Fig. 2A. These results suggest that HOXA10 may be phosphorylated by MEKK4, which in turn may influence the transcriptional regulation of downstream target genes by HOXA10 via homeoboxes.

### MEKK4 promotes embryo adhesion

**Fig. 3:**
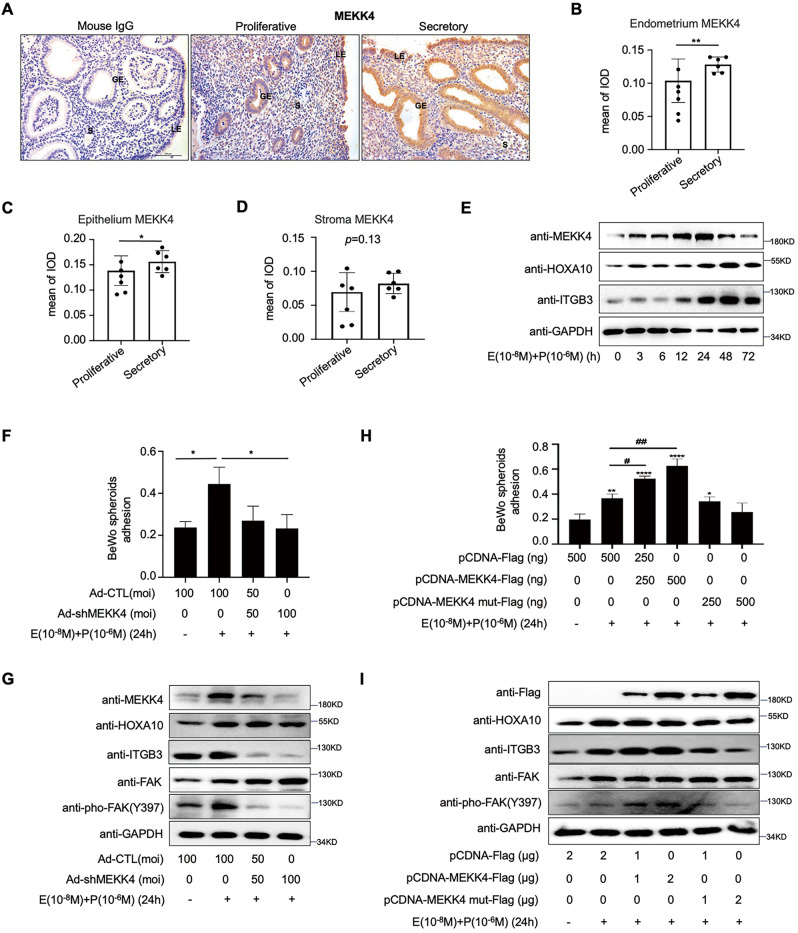
MEKK4 is mainly expressed in the endometrial epithelium and promotes embryo adhesion. **A**–**D** Immunohistochemical analysis of the MEKK4 expression in proliferative (*n* = 6) and secretory (*n* = 6) endometria from normal fertile women. Mouse IgG was used as a negative control. Data are represented as mean ± SEM, *P < 0.05, **P < 0.01, using *t*-test. (Scale bar = 100 µm). **E** Western blotting analysis of MEKK4, HOXA10, and ITGB3 expression in Ishikawa cells treated with E_2_ and MPA as indicated. **F** In vitro adhesion experiments analysis of the effect of MEKK4 knockdown on BeWo adhesion attached to the monolayer of Ishikawa cell. Values represent the mean ± SEM (*n* = 3); *P < 0.05, using one-way ANOVA. **G** Western blotting analysis of MEKK4, HOXA10, ITGB3, FAK, and pho-FAK expression in Ishikawa cells treated with the adenovirus Ad-shMEKK4 and then stimulated with E_2_ and MPA for 24 h. **H** In vitro adhesion experiments analysis of the effect of MEKK4 overexpression and kinase inactivation on BeWo adhesion attached to the monolayer of Ishikawa cell. Values represent the mean ± SEM (*n* = 3); ****P < 0.0001; **P < 0.01; *P < 0.05 compared with the pCDNA-Flag group; ##*P* < 0.01; #*P* < 0.05, using one-way ANOVA. **I** Western blotting analysis of MEKK4, HOXA10, ITGB3, FAK, and pho-FAK expression in Ishikawa cells transfected with MEKK4-Flag and MEKK4 mut-Flag (K1372R, kinase inactivation) and then treated with E_2_ and MPA for 24 h.

Immunohistochemical results displayed that MEKK4 expression in the secretory endometrium was obviously higher than that in the proliferative phase, and this phenomenon was mainly observed in the epithelial components of the endometrium (Fig. 3A–D). Combined stimulation with estrogen and progesterone significantly promoted the expression of the MEKK4 protein, with the most pronounced increase at 24 h (Fig. 3E). When the expression of endogenous MEKK4 was inhibited, the adhesion rate of BeWo cell spheroids was significantly reduced (Fig. 3F). In addition, MEKK4 promoted BeWo cell spheroids adhesion onto the monolayer of Ishikawa cells, and this promoting effect was abolished when MEKK4 kinase activity was lost (K372R) (Fig. 3H). Western Blot analysis further showed that the expression of the adhesion molecule ITGB3 and the activation of its downstream pathway was impaired after reduced expression or kinase inactivation of MEKK4 in Ishikawa cells (Fig. 3G, I).

**Fig. 4:**
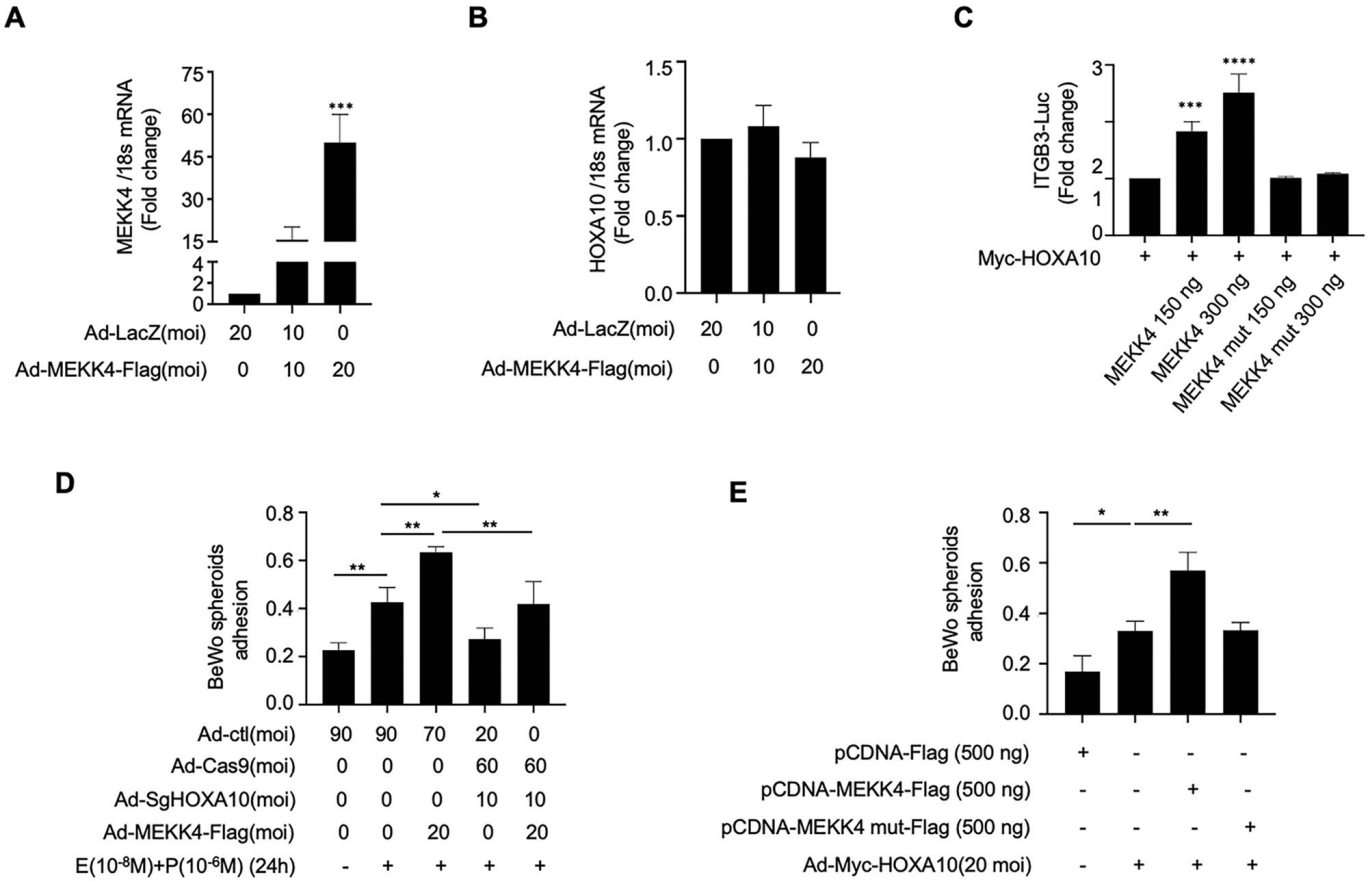
MEKK4 kinase activity influences HOXA10-promoted embryo adhesion. **A**, **B** Real-time PCR analysis of MEKK4 and HOXA10 mRNA levels in Ishikawa cells infected with the adenovirus Ad-MEKK4-Flag for 48 h. Data are represented as mean ± SEM, ***P < 0.001, using one-way ANOVA. **C** Luciferase reporter assay analysis of the ITGB3-Luc activities of Ishikawa cells transfected with MEKK4-Flag, MEKK4 mut-Flag, Myc-HOXA10 (150 ng), and ITGB3-Luc (150 ng) for 48 h. Data are represented as mean ± SEM, ****P < 0.0001, ***P < 0.001, using one-way ANOVA. **D** HOXA10 mediated MEKK4-promoted BeWo adhesion. Values represent the mean ± SEM (*n* = 3); **P < 0.01; *P < 0.05, using one-way ANOVA. **E** MEKK4 promoted HOXA10-induced BeWo adhesion, depending on its kinase activity. Values represent the mean ± SEM (*n* = 3); **P < 0.01; *P < 0.05, using one-way ANOVA.

The qPCR results showed that MEKK4 did not affect HOXA10 mRNA expression (Fig. 4A, B). High expression of MEKK4 significantly enhanced the transcriptional regulation of ITGB3 by HOXA10, and this potentiation disappeared after MEKK4 kinase inactivation (Fig. 4C). The promotion of embryo adhesion by MEKK4 was obviously weakened in Ishikawa cells with low HOXA10 expression (Fig. 4D), suggesting that the promotion effect was mediated by HOXA10. In addition, this enhancing effect of MEKK4 on HOXA10-promoted embryo adhesion was reduced after kinase inactivation (Fig. 4E), indicating that this effect of MEKK4 relies on its kinase activity.

### MEKK4 enhances HOXA10-promoted embryo adhesion through phosphorylating Thr362 of HOXA10

**Fig. 5:**
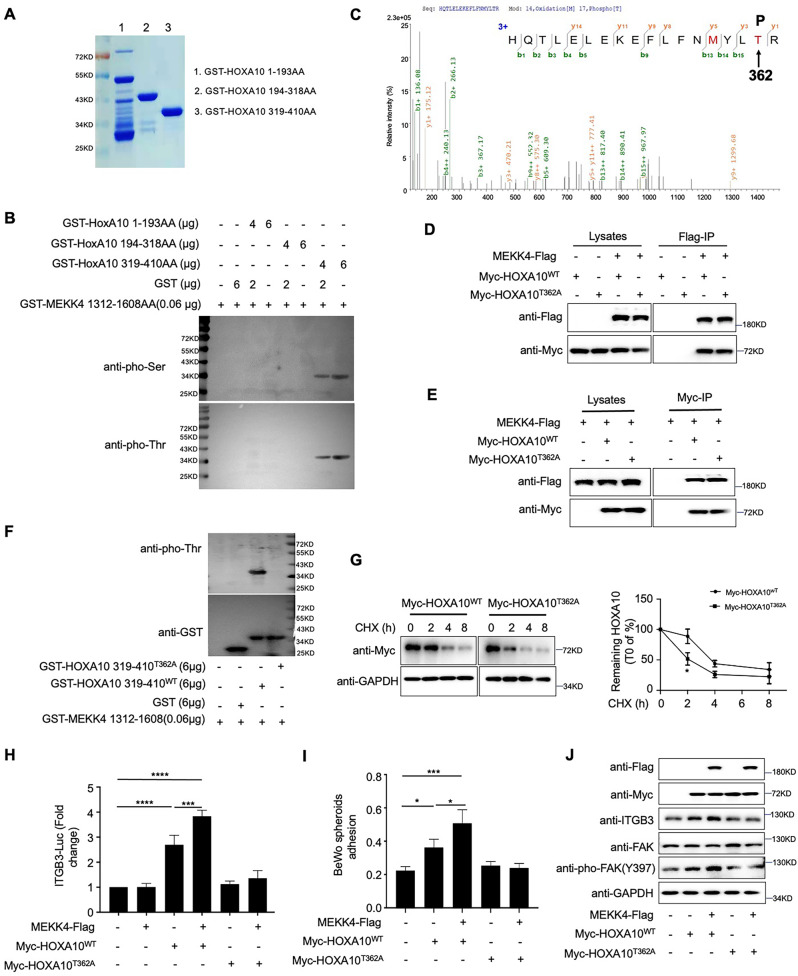
MEKK4 phosphorylates HOXA10 at T362 and affects the function of HoxA10. **A** Prokaryotic protein purification of various GST-HOXA10 mutant protein constructs (Coomassie blue staining). **B** MEKK4 phosphorylated various HOXA10 homeobox mutant constructs according to in vitro kinase assays. The solutions were analyzed by western blotting. **C** GST-HOXA10 319-410 was examined through mass spectrometry after SDS-PAGE electrophoresis by Thomas Brilliant Blue staining. **D**, **E** HOXA10 mutation did not affect the interaction between HOXA10 and MEKK4. **F** MEKK4 did not phosphorylate GST-HOXA10 319-410^T362A^ according to in vitro kinase assay. **G** 50 μg/ml CHX was added to the Ishikawa cells stimulated with Myc-HOXA10^T362A^ or Myc-HOXA10^WT^ for 2, 4, and 8 h. Western blot analysis of the levels of remaining HOXA10 in the cell extracts. The plots were relative to the levels at the 0 h time point. Values represent the mean ± SEM (*n* = 2); *P < 0.05 versus HOXA10^WT^ alone, using one-way ANOVA. **H** Luciferase reporter assay analysis of the ITGB3-Luc activities of Ishikawa cells transfected with MEKK4-Flag (300 ng), Myc-HOXA10^WT^ (150 ng), Myc-HOXA10^T362A^ (150 ng), and ITGB3-Luc (150 ng) for 48 h. Values represent the mean ± SEM (*n* = 3); ****P < 0.0001; ***P < 0.001, using one-way ANOVA. **I** After the HOXA10 mutation, the effect of MEKK4 on enhancing HOXA10-promoted BeWo adhesion was reduced. Values represent the mean ± SEM (*n* = 3); *P < 0.05; ***P < 0.001, using one-way ANOVA. **J** Western blotting analysis of Flag, Myc, ITGB3, FAK, and pho-FAK expression in Ishikawa cells stimulated with MEKK4-Flag, Myc-HOXA10^WT^, and Myc-HOXA10^T362A^ for 48 h.

To test whether MEKK4 directly phosphorylates HOXA10, we purified truncated prokaryotic protein HOXA10 fragments (Fig. 5A) and incubated them with active human MEKK4 recombinant protein. Western Blot showed that the HOXA10 319-410 peptide fragment could be phosphorylated by MEKK4 (Fig. 5B). We subsequently determined the HOXA10 phosphorylation sites by the mass spectrometry analysis. HOXA10 was phosphorylated at threonine 362 by MEKK4 (Fig. 5C). The mutation of threonine (Thr) to alanine (Ala) prevented phosphorylation at this site, and we subsequently generated a HOXA10 mutant, threonine (T) 362-to-alanine (A; T362A) mutant. The results displayed that the mutation of this site did not affect the association with MEKK4 (Fig. 5E) but affected the phosphorylation of HOXA10 by MEKK4 (Fig. 5F), implying that MEKK4 binds to HOXA10 and then phosphorylates HOXA10 directly through this site.

After the mutation of the HOXA10 active site threonine (HoxA10^T362A^), the protein stability of HOXA10 was decreased (Fig. 5G), its transcriptional regulation of the downstream gene ITGB3 was weakened (Fig. 5H), and its role in promoting embryo adhesion was reduced (Fig. 5I). In addition, the effect of MEKK4 on enhancing HOXA10-mediated transcriptional regulation and HOXA10-promoted embryo adhesion was also reduced (Fig. 5H, I). Western blotting verified that the ITGB3 signaling pathway was not significantly activated after the T362A mutation was introduced to HOXA10 (Fig. 5J). These results suggest that MEKK4 enhances the function of HoxA10 by phosphorylating Thr362.

### Aberrant low expression of MEKK4 in the endometrial epithelium of RIF patients

**Fig. 6:**
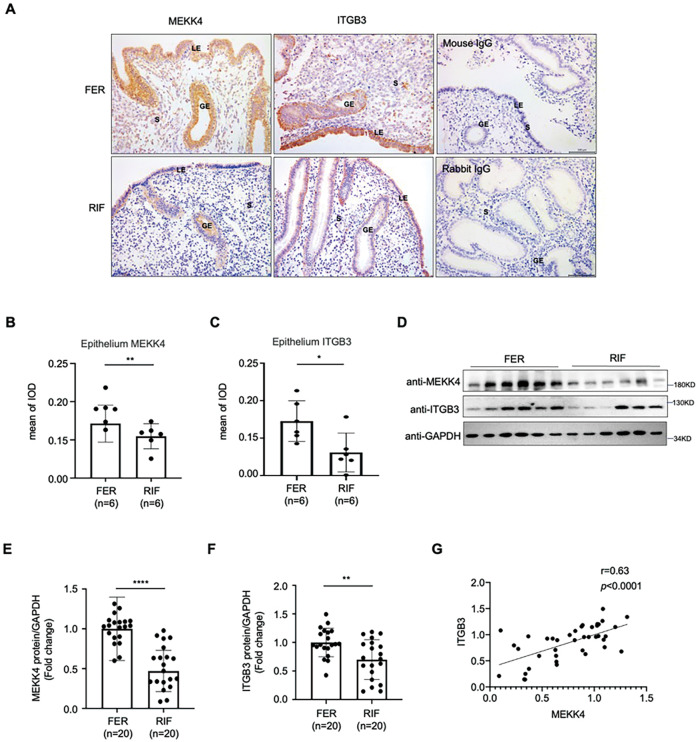
Aberrant low expression of MEKK4 in the endometria of RIF patients. **A**–**C** Immunohistochemical analysis of the MEKK4 and ITGB3 expression in the endometrial epithelia of fertile women (*n* = 6) and RIF patients (*n* = 6). The negative controls (NC) were mouse IgG and rabbit IgG. (Scale bar = 100 µm, Data are represented as mean ± SEM, **P < 0.01, *P < 0.05, using *t*-test). **D**–**F** Western blotting and quantitative densitometry analysis for MEKK4 and ITGB3 in the endometria of RIF patients (*n* = 20) and controls (*n* = 20). Data are represented as mean ± SEM, ****P < 0.0001; **P < 0.01, using *t*-test. **G** Correlation between MEKK4 and ITGB3 protein expression in the endometria of RIF patients and the FER group (*r* = 0.63, ****P < 0.0001, using simple linear regression and Pearson correlation coefficient).

We had seen that MEKK4 protein abundance and the ITGB3 protein abundance were lower in the endometria of RIF patients, especially in the epithelium of endometria by Immunohistochemical staining (Fig. 6A–C). Furthermore, the Western Blotting results also showed that MEKK4 and ITGB3 protein levels were obviously decreased in the endometria of RIF patients (Fig. 6D–F), and the expression of MEKK4 presented a moderate positive correlation with HOXA10 expression (*r* = 0.63, *P* < 0.0001) (Fig. 6G).

## Discussion

**Fig. 7:**
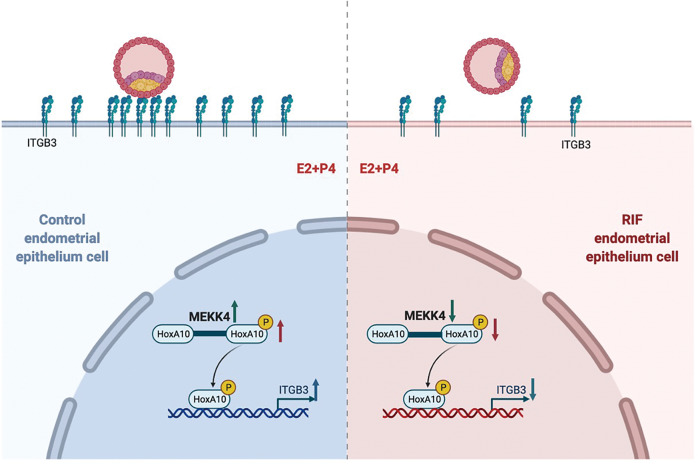
Schematic representation of the role of MEKK4 in the regulation of embryo adhesion in the fertile control groups and RIF patients. During the window of implantation, MEKK4 expression was elevated under estrogen and progesterone treatment, and the kinase phosphorylated HOXA10, which in turn transcriptionally activated ITGB3 to promote embryo adhesion. This effect decreased when MEKK4 expression was reduced, which might be a novel mechanism for implantation failure in RIF patients.

The implantation of a healthy embryo into the receptive endometrium is a critical step in establishing pregnancy; however, this step is considered a “black box” of infertility, and little is known about the underlying mechanisms of endometrial receptivity, the adhesion stage of implantation or how it is disturbed in infertile women [10]. Current research is mainly focused on the regulation of estrogen and progesterone signaling as well as the transcriptional regulation of various transcription factors and proteomics [6]. This study showed that HOXA10, a marker of endometrial receptivity, could be phosphorylated by the serine/threonine kinase MEKK4 at the threonine residue 362 of HOXA10. The mutation of this site affected both the transcriptional regulation of HOXA10 and HOXA10-promoted embryo adhesion. Fig. 7

The kinase MEKK4 is a member of the MAPK signaling pathway. The inactivation or knockdown of MEKK4 inhibits the activation of the p38 and JNK pathways in mouse neuroepithelial cells and thus increases apoptosis, leading to defective neural tube development in mice [24, 28]. Reduced phosphorylation of heat shock protein 27 (HSP27), a downstream molecule of p38, reduces skeletal protein stability, leading to abnormal skeletal development in mice [28]. MEKK4 also enhances SRY and SOX9 gene expression through the activation of p38 and GATA4, promoting mouse testis development and sex differentiation in fetal mice [25, 29]. It follows that the classical way in which MEKK4 functions is by activating the p38/JNK pathway. While the study showed that MEKK4 localized to the cytoplasm [23], we observed that MEKK4 was mainly located in the nucleus of the endometrial epithelium and was colocalized with the transcription factor HOXA10, a marker of endometrial receptivity. MEKK4 interacted with HOXA10 to promote the transcriptional regulation of HOXA10 on downstream target genes and enhance HOXA10-promoted embryo adhesion, suggesting that MEKK4 can directly interact with transcription factors and thus enhance their effects. Such intranuclear kinases have been observed to interact with transcription factors; for example, the intranuclear protein tyrosine phosphatase Shp2 interacts with ERα and then phosphorylates the Tyr residue of ERα, thereby promoting the transcriptional regulation of downstream target genes by ERα [30]. In addition, MEKK4 expression was higher in the receptive endometrium and decreased in the secretory endometrium of RIF patients. Thus, this study presented for the first time that MEKK4 functions in a nonclassical manner in the endometrial epithelium functions and that the HOXA10 protein can undergo phosphorylation modifications. Subsequently, we used phosphorylated antibodies to evaluate the endometria of RIF patients to further confirm whether p-HOXA10 could be used as an indicator for assessing endometrial receptivity.

The identification of HoxA10 protein phosphorylation modifications has mainly focused on myeloid leukemia cells thus far [31–33], and these modifications are even less studied in the uterus. The phosphorylation of tyrosine Y326 and Y343 in the homeobox of HOXA10 induces HOXA10 folding, which prevents homeobox binding to DNA or leads to the formation of HOXA10 homodimers that are unable to participate in DNA binding and thus act as transcriptional repressors [34]. A search of the phosphosite database revealed 10 HOXA10 protein phosphorylation sites (S19, S93, S272, T277, S313, S335, Y343, T348, and Y360), among which the Y343 site has been identified to act as a transcriptional repressor, leading to leukemogenesis [34]. The present study presented that the phosphorylation of T362 had a vital effect on the function of the HOXA10 protein and that the mutation of this site reduced the stability of the HOXA10 protein, the transcriptional regulation of downstream target genes, and the promotion of embryo adhesion. However, we focused on serine/threonine phosphorylation in this study, and then one could concern about the role of the phosphorylation of other amino acids, such as tyrosine phosphorylation, which could be considered in further studies to enrich the phosphorylation study of HOXA10 on the endometrium.

Phosphorylation modifications of many proteins play an important role in endometrial receptivity and embryo implantation. For example, estrogen and progesterone receptors are phosphorylated [30, 35]. During cAMP and MPA-induced decidualization, the differential regulation of the mTOR complex (mTORC1 activation and mTORC2 inactivation) results in reduced phosphorylation of Akt (S473), which further reduces FOXO1 (S256) phosphorylation. FOXO1 transcriptional activity in turn enhances the induction of metaphase marker genes (PRL, IGFBP1, WNT4, and LEFTY2) [36]; Stat3 phosphorylation is also important for embryonic adhesion [37] and decidualization [38]. There has been much research focused on specific signature molecules, and the lack of specificity among inhibitors and activators of the corresponding pathways has led to the emergence of more specific peptide therapeutics. For example, Stat3 peptide inhibitors have been developed [39]; the Stat3 SH2 structural domain binding peptide PYLKTK (Y means phosphor-tyrosine) competitively inhibits and disrupts the dimerization of Stat3. In vitro and in vivo experiments have demonstrated that Stat3 peptide inhibitors reduce the level of phosphorylated Stat3 in the epithelium in response to LIF, thereby reducing the induction of LIF-responsive genes, such as biallelic proteins and Irg1 [37]. In the future, we could subsequently design experiments targeting Thr362 of HOXA10 to identify specific peptide therapeutic agents to enhance the therapeutic effect of RIF.

The most comprehensive analytical tool available for phosphorylated proteins is phosphoproteomics. Phosphoproteomics, which includes the identification, localization, and quantification of phosphorylation, belongs to a branch of proteomics based on phosphorylation that is now increasingly being studied, as significant discoveries have been made in various fields related to phosphoproteomics [40]. An article published in Nature in 2019 described a study in which phosphoproteomics was combined with proteomics, transcriptomics, and whole-genome sequencing to find new therapeutic targets for early-stage hepatocellular carcinoma [41]; this study identified avasimibe, a sterol O-acyltransferase 1 (SOAT1) inhibitor, as a potential personalized treatment for patients. However, there are few phosphoproteomics studies in reproductive medicine. Hence, we can focus on the phosphorylation proteomics of the endometrium in physiological and pathological states to propose more precise research directions.

In conclusion, this study firstly reports that the human serine/threonine kinase MEKK4 protein interacts directly with HOXA10 and that this interaction causes phosphorylation modifications of HOXA10 at threonine 362. MEKK4 relies on its kinase activity to enhance HOXA10-promoted embryo adhesion, and MEKK4 is aberrantly expressed at low levels in the endometria of RIF patients. In addition, mutation of the HOXA10 phosphorylation site inhibits embryo adhesion. These observations suggest that HOXA10 phosphorylation is important for embryo implantation and could be a new focus for studies on the evaluation and treatment studies of RIF patients.

## Materials and methods

### Patient samples

The endometrial tissues were obtained from women attending the Center for Reproductive Medicine of Nanjing Drum Tower Hospital (approval from the ethics committee, 2013-408 081-01) in this study. All patients provided written informed consent. Proliferative endometria were obtained from 6 normal fertile females. Secretory endometria were obtained from 20 normal fertile females and 20 females with RIF. The normal group was comprised of women whose infertility was due to male factors and who were confirmed to be fertile after IVF-embryo transfer (IVF-ET) treatment. RIF was defined as failure to achieve pregnancy after the transfer of at least four good-quality cleavage-stage embryos or no less than two good-quality blastocysts over a minimum of two consecutive fresh or frozen cycles [5]. These groups showed no difference in age, and body mass index (Table 1). Patients with endometriosis, adenomyosis, endometrial hyperplasia, endometrial polyps, polycystic ovarian syndrome (PCOS), or hydrosalpinx were excluded.

**Table 1:** Demographic details of the participants in the study of endometrial MEKK4, HOXA10 and ITGB3 expression.

	FER(*n* = 20)	RIF(*n* = 20)	*P*-value
**Age**	30.57 ± 5.15	31.12 ± 4.06	NS
**Body mass index (kg/m**^**2**^**)**	22.12 ± 3.43	22.02 ± 2.64	NS
**No. of embryo transplantation**	1.85 ± 0.67	5.65 ± 1.66	<0.0001

NS = nonsignificant; *P* < 0.05 was considered significant.

### Cell culture

The Ishikawa cells are well-differentiated human endometrial adenocarcinoma cell lines that exhibit characteristics similar to endometrial tubular and glandular epithelial cells due to the presence of functional steroid receptors along with apical adhesiveness, making them suitable for studying embryo adhesion [42]. The cells were cultured in DMEM (Gibco) containing 10% fetal bovine serum (FBS, Clark) and 1% penicillin and streptomycin, at 37 °C in a humidified environment with 5% CO_2_. Under physiological conditions, the endometrium is affected by estrogen and progesterone and enters the secretory phase before reaching the receptive state. To simulate physiological conditions, the Ishikawa cells were treated with 10^−8^M E_2_ and 10^−6^M MPA (Sigma) with 2.5% charcoal/dextran-treated FBS (Gibco) in phenol red-free DMEM/F12 (Gibco) [20]. 50 μg/ml Cycloheximide (CHX) (Sigma) was performed in the protein degradation assay.

Ishikawa cell, BeWo cell, and 293 T cell were purchased from the Cell Bank of the Chinese Academy of Sciences. The cell lines were authenticated by STR profiling and tested negative for mycoplasma contamination.

### Immunohistochemical staining

Immunohistochemistry was performed on the human endometrium samples. These tissues were fixed with formalin solution overnight, dehydrated in alcohol, embedded in paraffin, and then sliced and mounted on slides. After dewaxing the tissue sections, endogenous peroxidase activity was blocked with 3% H_2_O_2_/PBS for 10 min. The slides were then autoclaved at 121 °C for 15 min in sodium citrate buffer (pH 6.0) for antigen retrieval. After cooling, the samples were blocked in goat serum for 30 min and then incubated with antibodies (MEKK4, 1:200, sc-100396, Santa Cruz; HOXA10,1:300, BA09181-1, Boster; ITGB3, 1:500, BS3660, Bioworld) at 4 °C overnight in a humidity chamber. As a negative control, the tissue sections were similarly pretreated and combined with the nonspecific rabbit or mouse IgG as the replacement for the primary antibody. The next day, the sections were hatched with goat anti-mouse or anti-rabbit secondary antibody at room temperature for 30 min and then counterstained with hematoxylin after diaminobenzidine (Invitrogen, Carlsbad, CA, USA) staining. Finally, Image-Pro Plus software was utilized to calculate the integrated optical density (IOD) of the immunohistochemical images.

### Coimmunoprecipitation and western blotting

293 T cells were transiently cotransfected with plasmids indicated. Forty-eight hours after transfection, the cells were lysed with a whole-cell lysing reagent (50 mM Tris-HCl pH 7.6, 1.0% NP-40, and150 mM NaCl) containing a protease inhibitor cocktail and phosphatase inhibitor cocktails 2 and 3 (Sigma). The concentrations of protein were determined via the Bradford assay (Bio-Rad). Next, 500 μg cell lysates were mixed with protein A/G PLUS-agarose beads (Abmart) at 4 °C for 2 h. Then, the lysates were mixed with 30 μl anti-Myc beads (Invitrogen) or 30 μl anti-Flag M2 beads (Sigma) at 4 °C overnight. The samples were subjected to SDS-polyacrylamide gel electrophoresis (PAGE), transferred to PVDF membranes (Millipore), and analyzed by western blotting using an anti-Flag-HRP monoclonal antibody (1:10 000, Sigma) and an anti-Myc HRP-conjugated antibody (1:10 000, Invitrogen).

The Ishikawa cells were lysed with whole-cell lysis buffer. Equal amounts of total proteins (20–50 μg) were separated on 10% SDS-polyacrylamide gels and transferred to PVDF membranes (Millipore). The membranes were then blocked with Tris-buffered saline solution containing 5% nonfat milk for 1 h, and further incubated with the following primary antibodies: MEKK4 (1:500; sc-100396, Santa Cruz), ITGB3 (1:1000; BS3660, Bioworld), pho-Ser (1:1 000; P3430, Sigma), pho-Thr (1:1 000; PT-5H5, Thermo), FAK (1:1 000; 12636-1-AP Abways), p-FAK (Y397) (1:1 000; 10357-1-AP, Abways), and GAPDH (1:10 000; AP0063, Bioworld), which served as an internal control. Immunodetection was performed with goat anti-rabbit or goat anti-mouse HRP conjugated secondary antibodies and accomplished with an enhanced chemiluminescence kit (Millipore).

### Luciferase reporter assay

The luciferase reporter plasmid ITGB3-Luc carrying the ITGB3 promoter was characterized previously [19]. Preconfluent (60%) Ishikawa cells in 24-well plates were transfected with the plasmids as indicated. And after 48 h, cells were analyzed for luciferase activities with a Dual-Luciferase Assay System (Promega). According to the manufacturer’s instructions, luciferase activity was measured using a luminescence counter (Berthold Technologies). Firefly luciferase activity was normalized to the corresponding Renilla luciferase activity to assess the transfection efficiency.

### Immunofluorescence staining

Ishikawa cells were washed with 1xPBS and fixed with 4% paraformaldehyde (w/v) for 30 min at indoor temperature. And then, the fixed coverslips were permeabilized with 0.1% Triton X-100/PBS for 5 min at indoor temperature after washing three times with PBS for 5 min each. Nonspecific sites were blocked with 3% BSA/PBS for 1 h at 37 °C. The cells were hatched with an anti-HOXA10 antibody (1: 200; Boster) and an anti-MEKK4 antibody (1: 200; Santa Cruz) at 4 °C overnight. The coverslips were further hatched with Alexa Fluor 594-conjugated donkey anti-rabbit IgG (1: 500, Invitrogen) and Alexa Fluor 488-conjugated goat anti-mouse IgG (1: 500, Invitrogen) after washing three times with PBS for 5 min each. Nuclei were stained with 4',6-diamidino-2-phenylindole dihydrochloride (DAPI) (Sigma).

### RNA isolation and qPCR

Total RNA was extracted using TRIzol Reagent (Life Technologies) following the standard manufacturer’s protocol. 1 µg of total RNA was reverse transcribed by using RT Master Mix (G492, ABM) to generate cDNA. Real-time PCR analysis of the MEKK4 and HOXA10 mRNA levels were quantified on an Analytik Jena instrument. The primer sequences are seen in Table 2.

**Table 2:** Primers.

Primer	Sequence
**RT-PCR**	
Human MEKK4-F	AAAGTCGTGCCTCAGGTGGAGA
Human MEKK4-R	CCTCAATGGACTGCTGGAAAGC
Human HOXA10 -F	AGGTGGACGCTGCGGCTAATCTCTA
Human HOXA10-R	GCCCCTTCCGAGAGCAGCAAAG
Human 18 S rRNA-F	CGGCTACCACATCCAAGGAA
Human 18 S rRNA-R	CTGGAATTACCGCGGCT
**PCR (plasmid construct)**	
GST-HOXA10 1-193AA-F	CCGGAATTCATGTCAGCCAGAAAGGGCTATCTGCT
GST-HOXA10 1-193AA-R	TATCTCGAGTCACCGCGGGAAGGGAGCCAGTT
GST-HOXA10 194-318AA-F	TATAGAATTCGGCCCGCCGCCCGACG
GST-HOXA10 194-318AA-R	ATACTCGAGTCAGGAATCCTTCTCCGGCGAGGCTT
pCS2-Myc-HOXA10 T362A-F	TATGTACCTTGCACGAGAGCGGCGCCTAGAGATT
pCS2-Myc-HOXA10 T362A-R	CGCCGCTCTCGTGCAAGGTACATATTGAACAGAA
pSilencer-CMV-MEKK4-F	GATCCGGCTGCTGAATTGCAGTTTAGGTTCAAG AGACCTAAACTGCAATTCAGCAGCCTTTTTTG
pSilencer-CMV-MEKK4-R	AATTCAAAAAAGGCTGCTGAATTGCAGTTTAGGA AGTTCTCTCCTAAACTGCAATTCAGCAGCCG

### Construction of plasmid and adenovirus vectors

GST-HOXA10 319-410AA, pCDNA-MEKK4-Flag, pCDNA-MEKK4-Flag K1372R, pEGFP-N1-MEKK4, and pFLAG-CMV-MEKK4 1-941AA were constructed by the company. The pCS2-Myc-HOXA10 plasmid and adenovirus Ad-myc-HOXA10 were previously constructed [19]. Various plasmids were constructed by using pCS2-Myc-HOXA10 and pCDNA3.1(+)-MEKK4-Flag expression plasmids as templates. These PCR products were cloned into the pGEX-4T-1 plasmid; A threonine-to-alanine mutant of HOXA10 T362A was generated via recombination methods to construct the pCS2-Myc-HOXA10 T362A and GST-HOXA10 319-410 T362A plasmids. These primers were designed as shown in Table 2.

Adenovirus vectors were generated using AdMax (Microbix) systems according to the manufacturer’s recommendations. Viruses were packaged and amplified in HEK293A cells and purified based on CsCl banding, followed by dialysis against 10 mmol/L Tris-buffered saline with 10% glycerol.

### Prokaryotic protein purification

The pGEX-4T1 constructs were subjected to BL21(DE3) competent cells (Vazyme) for transformation and then prokaryotic protein expression was induced with 0.2 mM IPTG (BioFroxx) at 37 °C for 3 h. After the bacterial precipitates were lysed and ultrasonically crushed, prokaryotic HOXA10 mutant proteins were purified by mixing the precipitates with Pierce Glutathione Agarose (Thermo). Finally, the beads were eluted with GSH (Sigma) overnight to obtain the purified protein.

### In vitro phosphorylation assay

Equal amounts of recombinant MEKK4 (Kinase activity, P5591, Abnova) were incubated with purified protein different fragments of HOXA10 in 25 µL kinase buffer (40 mM HEPES (pH 7.4), 20 mmol/L MgCl_2_, 1 mM DTT, and 10 µm ATP) for 60 min at 30 °C. Reactions were terminated by 5 × SDS sample buffer, and the products were loaded onto 10% SDS-PAGE gels. The phosphorylation of HOXA10 was detected by immunoblotting with anti-phospho-Ser and anti-phospho-Thr antibodies. Coomassie bright blue staining and tandem mass spectrometry were performed to detect phosphorylation sites.

### In vitro adhesion assay [43]

Ishikawa cells in a 24-well plate were stimulated as indicated. 80%-90% confluence of BeWo cells were digested to the cell suspension and then placed in a 60 mm^2^ dish coated with the anti-adhesive polymer, poly-2-hydroxyethyl methacrylate (P3932, Sigma), to inform 150–200 μm BeWo spheroids after 36 h culture. The spheroids were transferred to confluent Ishikawa cell monolayers. After incubation at 37 °C for 40 min, the unattached spheroids were removed by washing with PBS. The attachment rate was valued as the number of attached spheroids divided by the total number of spheroids added to the Ishikawa cells.

### PyBioMed predict the protein interaction [44]

According to the amino acid sequence and structure information of MEKK4 protein in the Uniprot database (https://www.uniprot.org/) and Alphafold database (https://alphafold.ebi.ac.uk/), we used the PyBioMed software package to predict the interaction between individual molecules of MEKK4 and HOXA10, using default parameters for all parameters. It was screened that MEKK4 had a strong interaction with HOXA10.

### Statistical analysis

The data are presented as the mean ± SEM of at least three independent experiments unless otherwise indicated. Prism version 8 software was used for statistical analyses. Student’s *t*-test (normal distribution) was conducted to test for the differences between the two groups. For experiments involving more than two groups, one-way ANOVA was used. *P* < 0.05 was considered statistically significant.

## Supplementary information

Original Data File

## Data Availability

All datasets generated and analyzed during this study are included in this published article. Additional data are available from the corresponding author on reasonable request.
