# Supplementary material for: MEKK4-mediated Phosphorylation of HOXA10 at Threonine 362 facilitates embryo adhesion to the endometrial epithelium
Source: Cell Death Discov. 2022 Oct 10;8:415. doi: 10.1038/s41420-022-01203-1 (PMC9550837; doi:10.1038/s41420-022-01203-1)
Supplement: Supplementary file 1 — Original Data File [file 41420_2022_1203_MOESM1_ESM.docx]

**Figure 1A， Figure 6D-G original picture (n=20 vs 20)**

**Endometrium FER(n=6) vs RIF(n=6)**

**
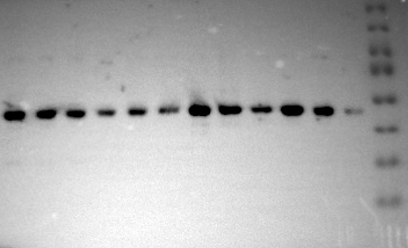
 anti- HOXA10 Figure1A**

**
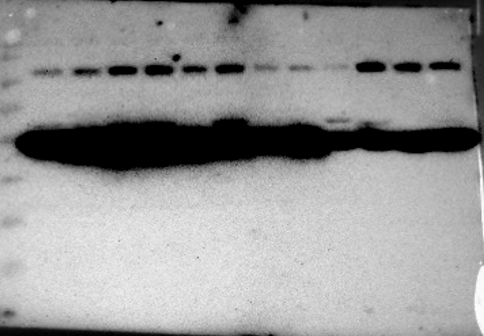
anti-ITGB3 Figure 6D**

**
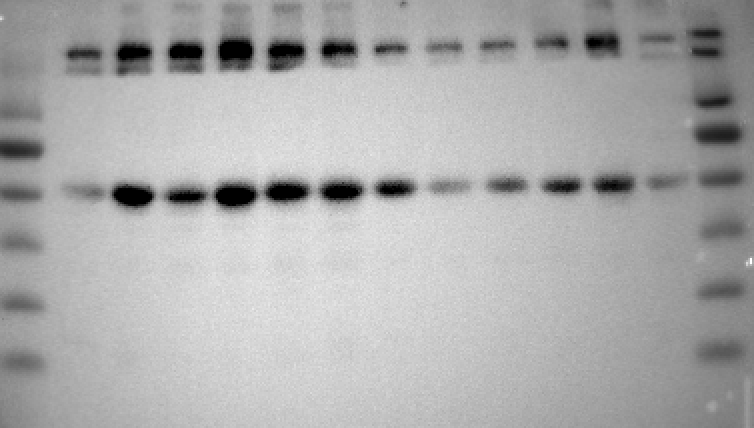
anti-MEKK4. Figure 6D**

**
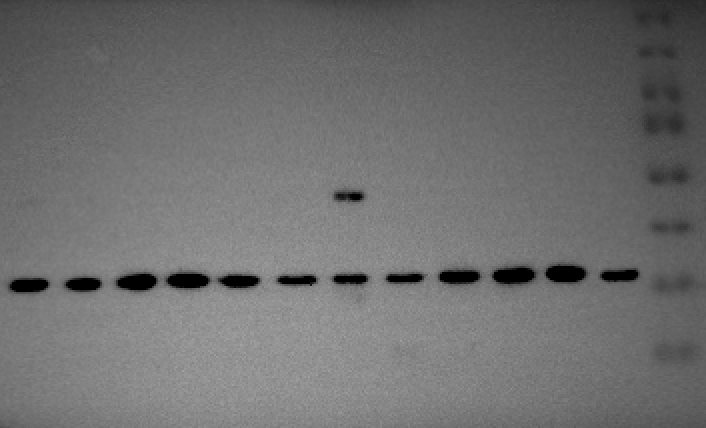
anti-GAPDH. Figure1A. Figure 6D**

**Endometrium FER(n=7) vs RIF(n=7)**

**
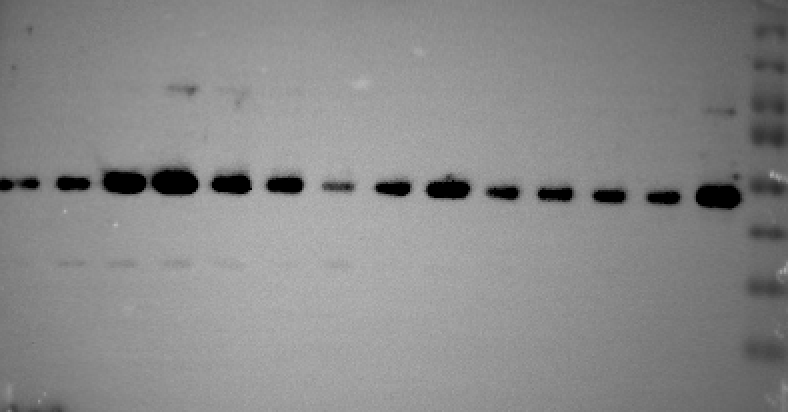
 anti- HOXA10**

**
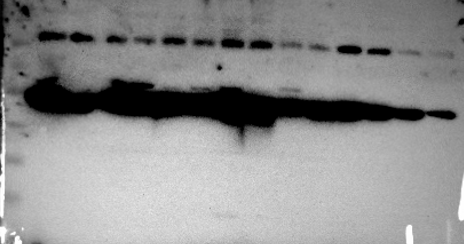
 anti-ITGB3**

**
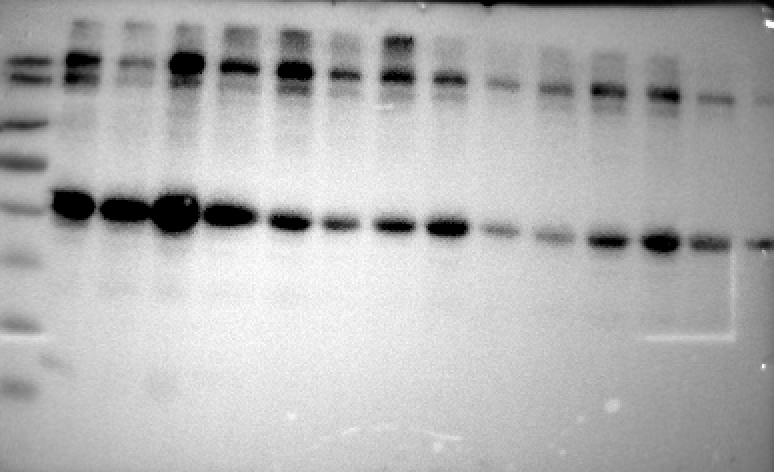
 anti-MEKK4**

**
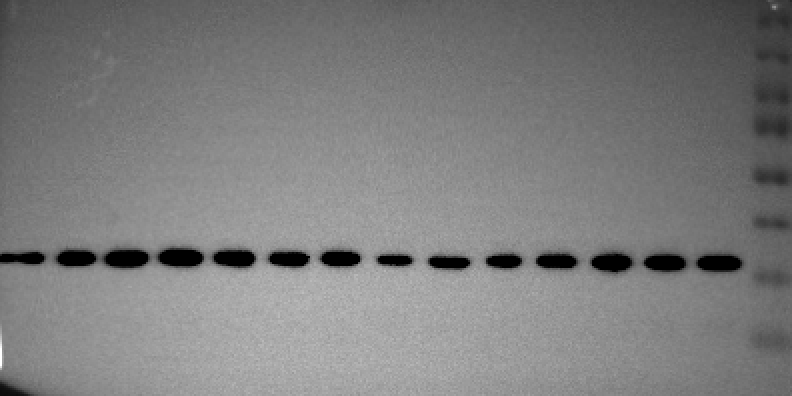
 anti-GAPDH**

**Endometrium FER(n=7) vs RIF(n=7)**

**
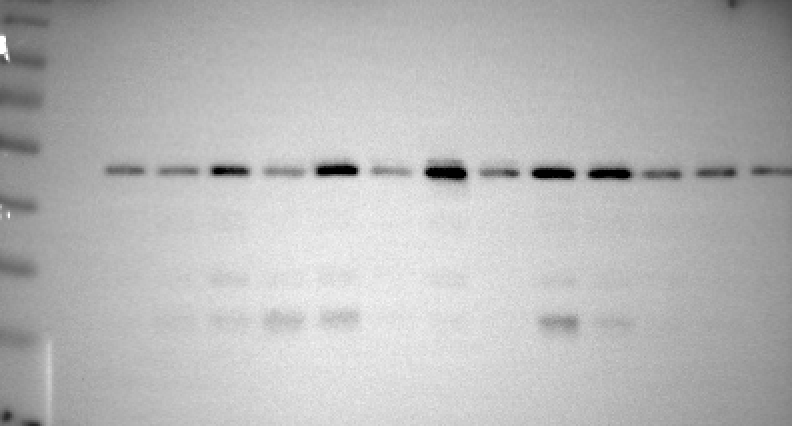
 anti- HOXA10**

**
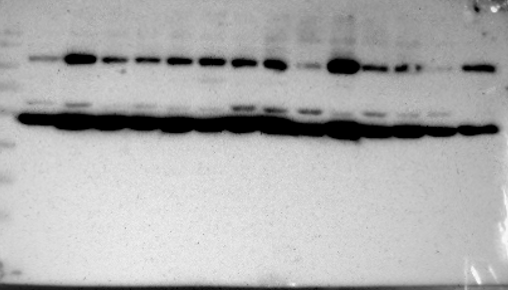
 anti-ITGB3**

**
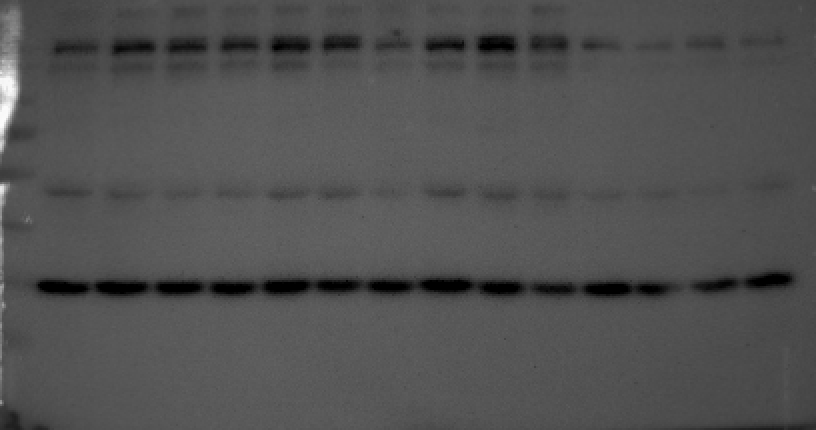
 anti-MEKK4**

**
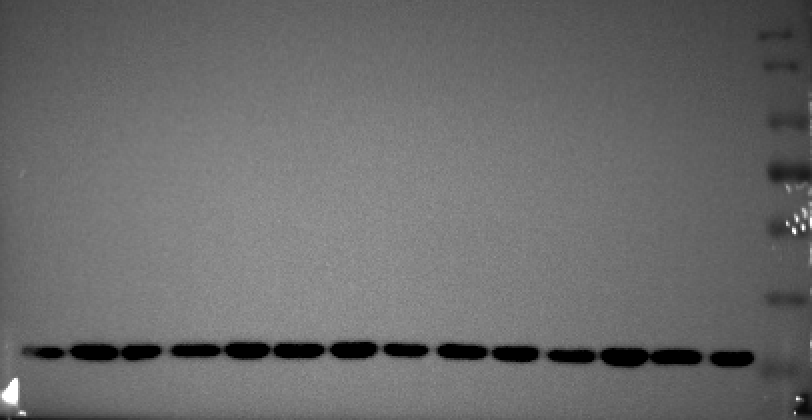
 anti-GAPDH**

**Figure 1C-E original picture (n=7 vs 7)**

**Endometrium FER(n=7) vs RIF(n=7)**

**
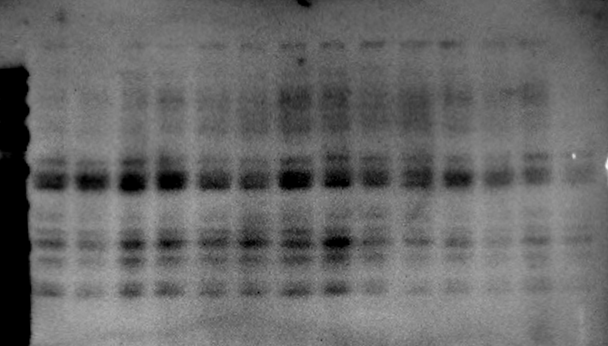
 anti-pho-Ser**


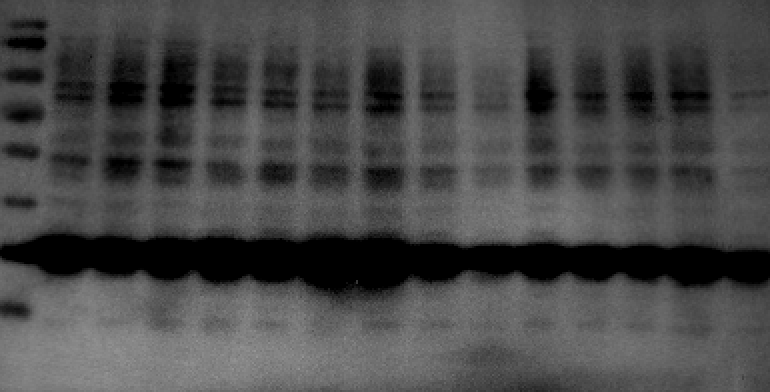
 **anti-pho-Thr**


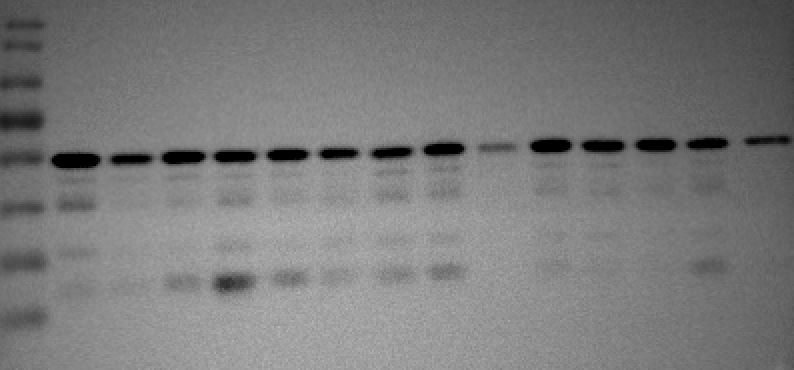
 **anti- HOXA10**


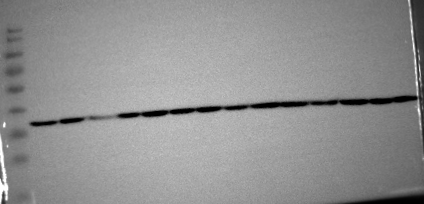
 **anti-GAPDH**

**Figure 1F original picture**


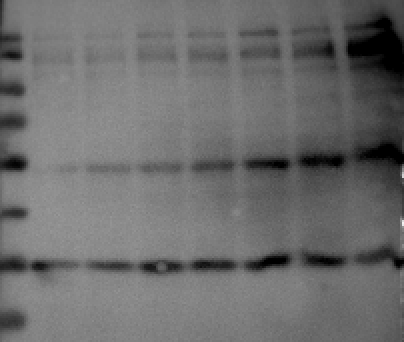
 **
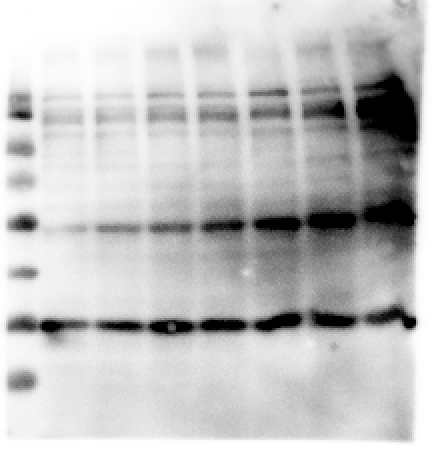
 anti-pho-Ser**
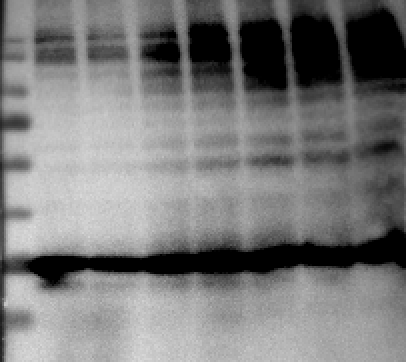
 **
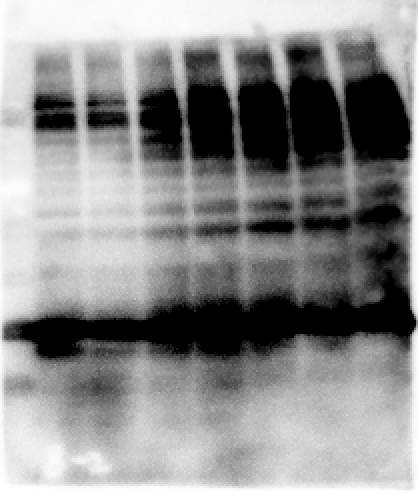
 anti-pho-Thr**

**Add white light background. no white light background +long exposure**


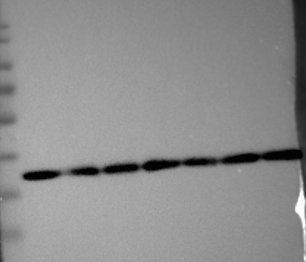
 **anti- GAPDH**

**Figure 1G original picture**


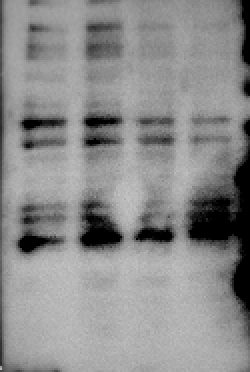
 **anti-pho-Ser**
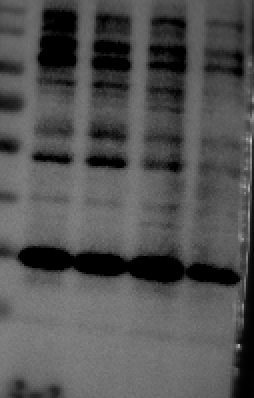
 **anti-pho-Thr**


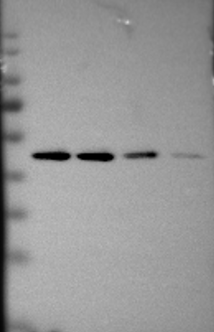
 **anti- HOXA10**
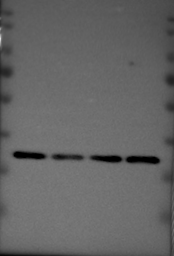
 **anti- GAPDH**

**Figure 1H original picture**


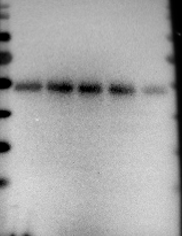

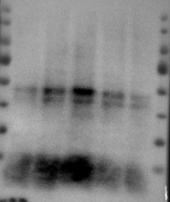

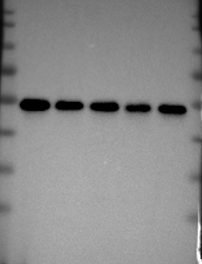


**anti-pho-Ser anti-pho-Thr anti- myc**

**Figure 2B original picture**


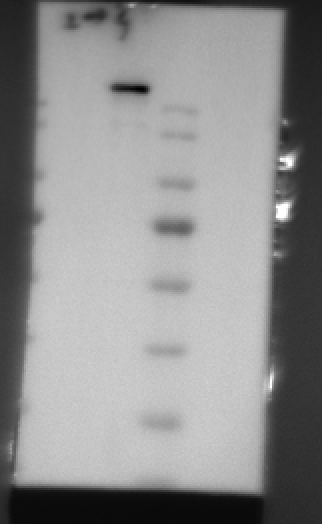

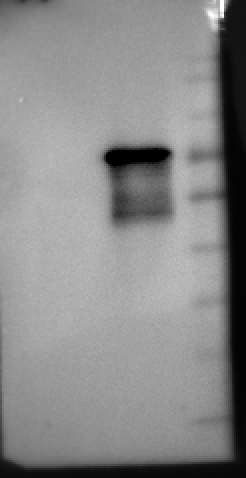
  **Myc-IP**

**anti-Flag anti-Myc**


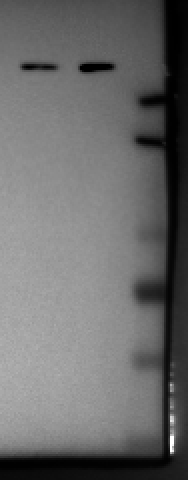

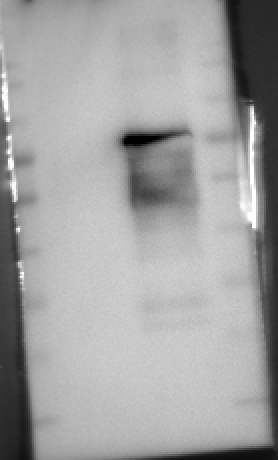
 **lysis**

**anti-Flag anti-Myc**

**Figure 2C original picture**


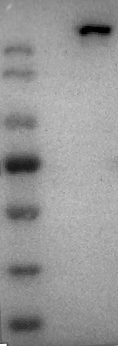

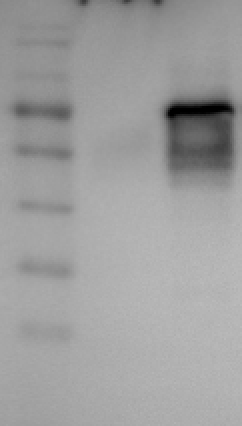
 **Flag-IP**

**anti-Flag anti-Myc**


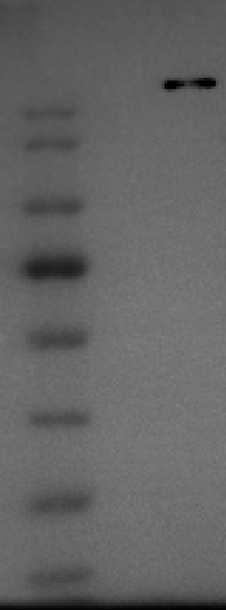

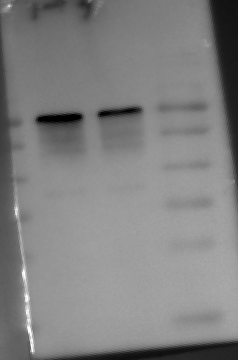
  **lysis**

**Figure 2D original picture**


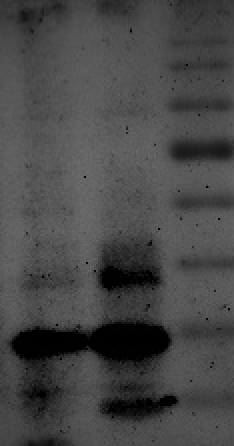

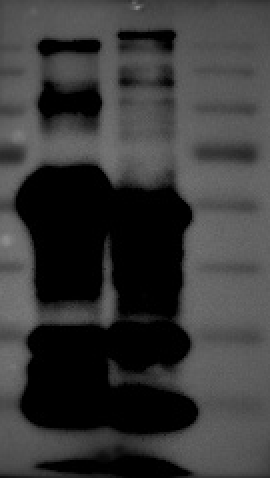


**anti-HOXA10 anti-MEKK4**

**Figure 2G original picture**


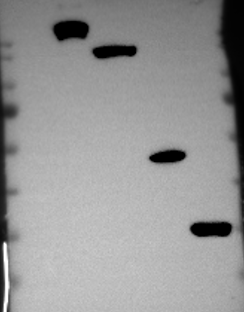

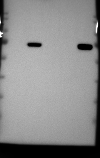
 **Flag-IP**

**Anti-Flag anti-Myc**


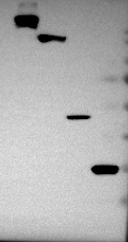

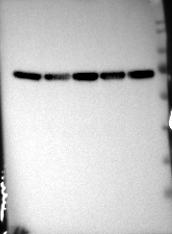
  **lysis**

**Figure 2H original picture**


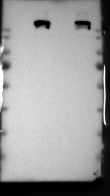

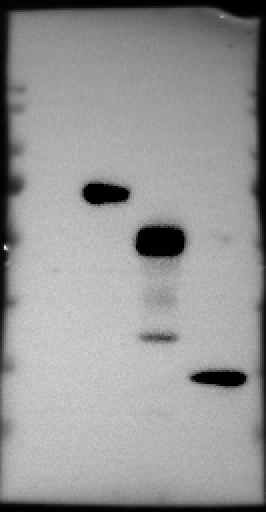
  **Myc-IP**

**Anti-Flag anti-Myc**


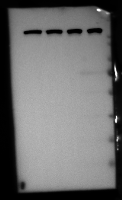

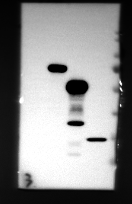
 **lysis**

**Figure 3E original picture**


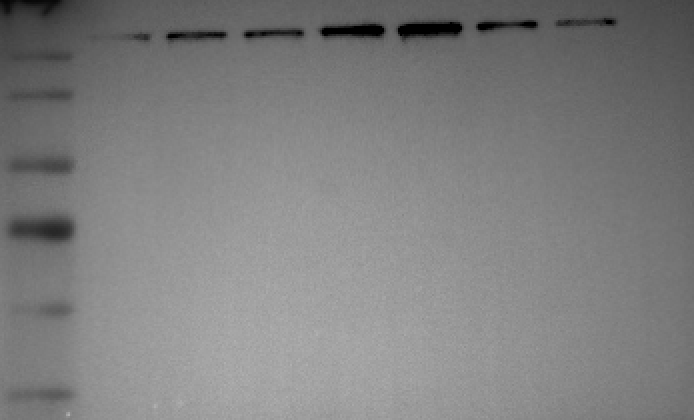

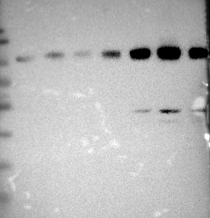


**anti- MEKK4 anti- ITGB3**


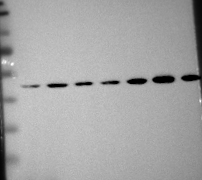

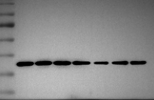


**anti- HOXA10 anti- GAPDH**

**Figure 3G original picture**


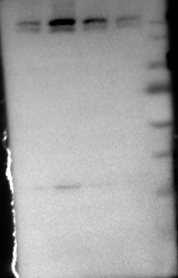
 **anti- MEKK4**
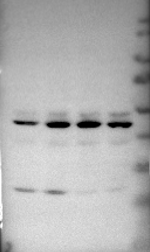
 **anti- HOXA10**


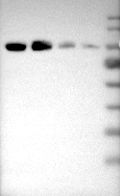
**anti- ITGB3**
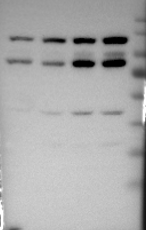
 **anti- FAK**


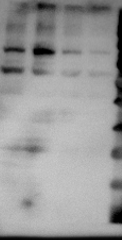
 **anti-p-FAK**
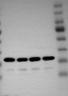
 **anti- GAPDH**

**Figure 3I original picture**


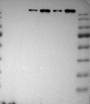
**anti- MEKK4**
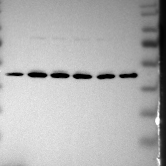
 **anti- HOXA10**


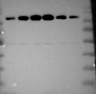
**anti- ITGB3**
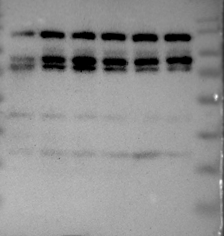
 **anti- FAK**


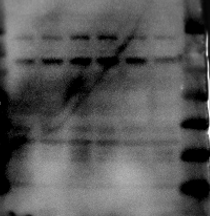
 **anti-p-FAK**
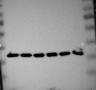
 **anti- GAPDH**

**Figure 5B original picture**

**
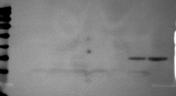
 anti-pho-Ser**

**
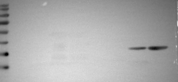
 anti-pho-Thr**

**Figure 5D original picture**


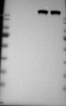

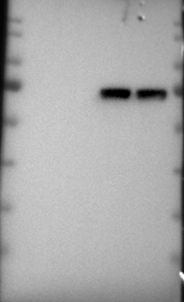
 **Flag-IP**

**Anti-Flag anti-Myc**


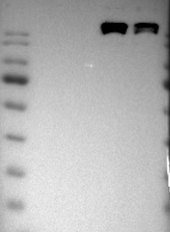

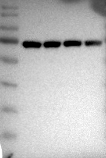
 **lysis**

**Figure 5E original picture**


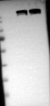

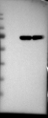
 **Myc-IP**

**anti-Flag anti-Myc**


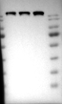

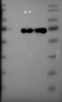
 **lysis**

**Figure 5F original picture**

**
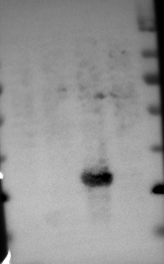

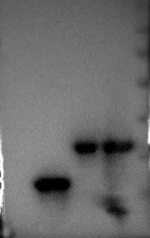
**

**anti-pho-Thr anti-GST**

**Figure 5G original picture**


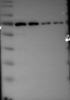

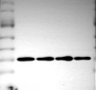
 **hoxa10 ^wt^**

**anti-Myc anti-GAPDH**


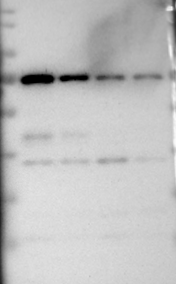

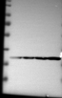
 **hoxa10 ^t362a^**


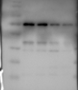

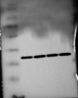
 **hoxa10 ^wt^**

**anti-Myc anti-GAPDH**


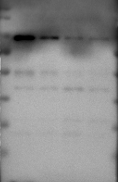

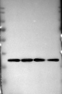
 **hoxa10 ^t362a^**

**Figure 5J original picture**


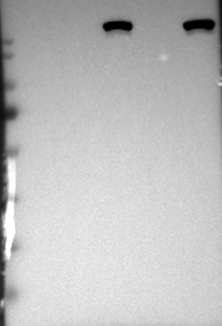
 **anti-Flag.**
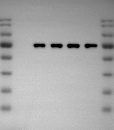
 **anti-Myc**


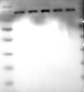
 **anti-ITGB3**
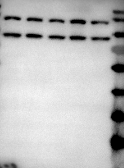
 **anti-FAK**


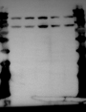
 **anti-p-FAK**
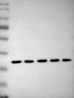
 **anti-GAPDH**
